# Nutritional status and adequacy of feeding Practices in Infants and Toddlers 0-23.9 months living in the United Arab Emirates (UAE): findings from the feeding Infants and Toddlers Study (FITS) 2020

**DOI:** 10.1186/s12889-022-12616-z

**Published:** 2022-02-15

**Authors:** Leila Cheikh Ismail, Ayesha S. Al Dhaheri, Sarah Ibrahim, Habiba I. Ali, Fatima Al Zahraa Chokor, Lynda M. O’Neill, Maysm N. Mohamad, Amira Kassis, Wafaa Ayesh, Samer Kharroubi, Nahla Hwalla

**Affiliations:** 1 Department of Clinical Nutrition and Dietetics, College of Health Sciences, University of Sharjah, Sharjah, 27272 United Arab Emirates; 2 Nuffield Department of Women’s & Reproductive Health, University of Oxford, Oxford, OX1 2JD UK; 3 Department of Nutrition and Health, College of Medicine and Health Sciences, United Arab Emirates University, Al Ain, 15551 United Arab Emirates; 4 Department of Nutrition and Food Sciences, Faculty of Agricultural and Food Sciences, American University of Beirut, P.O. Box 11-0236, Riad El Solh, Beirut, Lebanon; 5 Nestlé Institute of Health Sciences, Nestlé Research Center, Société des Produits Nestlé S.A., Vers-chez-les-Blanc, 1000, 26 Lausanne, Switzerland; 6 Whiteboard Nutrition Science, Beaconsfield, Quebec, Canada; 7 Public Health Protection Department, Dubai Health Authority, Dubai, United Arab Emirates

**Keywords:** United Arab Emirates, Infants and toddlers, Malnutrition, Stunting, Overweight, Obesity, Breastfeeding, Complementary feeding, Food intake

## Abstract

**Background:**

Infant and young child feeding practices (IYCF) impact the early and later health status of the population. Limited data is available regarding IYCF in the United Arab Emirates (UAE). This study aimed to evaluate the nutritional status and adequacy of feeding practices, energy, food, and nutrient intakes of UAE infants and toddlers ages 0-23.9 months.

**Methods:**

This study is a population-based cross-sectional survey of 276 infants and toddlers aged 0-23.9 months of which 180 were nationals and 96 were Arab non-nationals living in the UAE. Data were collected from the three major emirates: Abu Dhabi, Dubai, and Sharjah. Anthropometry was collected and assessed using WHO Anthro-Survey Analyzer, and a multicomponent age-specific questionnaire was used to evaluate IYCF and sociodemographic characteristics. Usual intake of energy, micronutrients, and macronutrients, including supplements, were collected using multiple-pass 24-h dietary recall and analyzed using the PC-side software. IYCF practices were assessed using the WHO indicators.

**Results:**

Overall, 4% of children were malnourished, 8% wasted, 15% stunted, 18% at risk of overweight, and 7% overweight and obese. 95% of infants were ever breastfed and 37% exclusively breastfed at 6 months. Around 98% of infants had a timely introduction of complementary food. Macronutrient intake exceeded AMDR for fat, carbohydrates, and protein for 27%, 8% and 2% of toddlers respectively. As for the percentage of those exceeding the ESPGHAN cut-off for free sugars set at 5% of energy intake, 28.6% had excessive intakes overall, 10% in 0-5.9, 21.9% in 6-11.9 and 56.7% in 12-23.9 month. Usual iron intake was below the Estimated Average Requirement (EAR) in 47% of infants 6-11.9 months. Above 12 months, the usual intake of iron and vitamin D were below EAR in 11% and 49% of toddlers respectively. Usual intake exceeded the tolerable upper intake levels (UL) for vitamin A (14 to 18%) and zinc (11 to 22%) across all ages. Approximately 93% of toddlers ages 12–23.9 months did not meet food groups’ recommendations for vegetables, 87% for fruits, 48% for milk/dairy, 54% for lean meat and beans, and 33% for grains.

**Conclusions:**

This study revealed that a high percentage of infants and toddlers aged 0-23.9 m suffer from a double burden of malnutrition, which is the coexistence of both undernutrition, and overweight and obesity in the same population. In addition to suboptimal feeding practices and inadequate/overconsumption of various nutrients. The findings highlight the need for the development of culturally specific programs aiming to improve the nutritional status of infants and toddlers in the UAE.

**Supplementary Information:**

The online version contains supplementary material available at 10.1186/s12889-022-12616-z.

## Background

The Middle East and North African (MENA) region is facing a triple burden of malnutrition including stunting, wasting, micronutrient deficiencies, as well as an escalating prevalence of obesity and associated non-communicable diseases (NCD) [1].

Nutrition transition and food consumption quality and practices have been emphasized to be driving malnutrition in these countries. The UAE is experiencing rapid economic growth with increased income, trade, and marketing along with rapid urbanization [2]. Along with these changes came a nutrition transition accompanied by an increased prevalence of obesity and metabolic diseases [3]. This nutrition transition in the UAE has been linked to a shift in dietary intake where traditional diet (rich in dairy products, fruits, and vegetables) is being replaced by a western diet characterized by higher fat, high sugar, refined foods [2]. Data on malnutrition and feeding practices of infants and toddlers are scarce in the region, and if available, would allow countries to design and implement evidence-based interventions and policies leading to improved nutritional status of the population. Early feeding practices and childhood overweight and obesity have been reported to be associated with a subsequent increase in the risk of later obesity and NCDs such as type 2 diabetes and cardiovascular diseases (CVD) [4]. The first 1,000 days of life, from conception to two years of age, is a critical window of opportunity that influences adequate growth and development and provides protection against both under and overnutrition in the short and long term [5, 6]. Early childhood feeding practices are believed to be the underlying factors that result in many of the Gulf Cooperation Countries (GCC) suffering from the highest levels of adult obesity and NCDs worldwide [7]. Moreover, childhood undernutrition (such as stunting and wasting), may result in increased child mortality, poor cognition, poor school performance, and poor earning potential in later life [8]. Identifying and tackling poor feeding practices of infants and young children is a crucial strategy to prevent malnutrition and alleviate chronic diseases.

WHO set international recommendations for infants and young children feeding practices (IYCF) calling for exclusive breastfeeding for six months and continued breastfeeding for twenty-four months or more [6]. Various studies have shown that exclusive breastfeeding for six months decreases the risk of later overweight and obesity, sudden infant death syndrome, respiratory illness, diarrhea, and the risk of developing NCDs [9–11]. Moreover, studies also revealed a higher IQ and academic achievement among children and adolescents who were exclusively breastfed for six months [11, 12].

In addition to exclusive breastfeeding, the WHO recommends the initiation of complementary feeding at six months of age as breastmilk alone will no longer meet the nutritional needs for the rapid growth of the child [13]. The introduction of complementary food is a critical transition phase that requires special attention to the provision of age-appropriate and nutritionally adequate food for optimal child growth and development [1]. A lower occurrence of diarrhea along with gastrointestinal infections and a better growth pattern was reported among children who had a delayed introduction of complementary food until six months of age [14–16].

Despite the growing evidence for the impact of early nutrition on future health outcomes, little data is available regarding IYCF feeding practices in Gulf countries. In fact, in the UAE, limited national data is available regarding the duration of exclusive breastfeeding, breastfeeding practices, and the onset and adequacy of complementary feeding. Few studies are available on selected emirates (Dubai or Abu Dhabi) [17, 18] and recent UNICEF report on state of world children shows that the figures for these parameters are not available [8].

This study aims to shed light on the nutritional status and feeding practices of infants and toddlers (ages 0 – 23.9 months) in three major UAE Emirates: Abu Dhabi, Dubai, and Sharjah. UAE enjoys a highly diverse population, comprised of approximately 9.89 millions of which 11% are UAE nationals and 89% non-UAE nationals [19]. Nutritional status, feeding practices, dietary intake, and meal patterns of infants and toddlers were assessed and potential differences in the IYCF practices between UAE nationals and Arab non-nationals living in the UAE were measured. The findings of the study will allow for the formulation of evidence-based interventions as well as nutrition-related policies for infants and young children in the UAE.

## Methods

### Study design

This study was designed to assess the anthropometry, dietary intake, and feeding practices of UAE nationals and Arab non-nationals’ infants and toddlers in line with the Feeding Infants and Toddler Study (FITS). The latter is a prototype (model) of dietary intake survey of a large cross-sectional sample of infants and toddlers aimed at building and sharing nutrition knowledge across 10 countries [20]. Data were collected from three emirates: Abu Dhabi, Dubai, and Sharjah which are home to approximately 85% of the population [21]. The study sample was recruited using a stratified random cluster sampling framework. Within this framework, the three UAE emirates constituted the various representative strata. These emirates consisted of many districts (regions), and neighbourhoods within districts made up of the various clusters. The number of clusters (neighbourhood) per district differed depending on the population density in that district. Clusters within Emirates were chosen based on probability proportional to size sampling. The total number of subjects selected from each Emirate was also proportional to the population. The sample size calculations for the UAE nationals were based on an estimated prevalence of obesity of 13% [22] with a 95% confidence interval (CI) and a margin of error of 5%. These calculations were carried out using the STEPS Sample Size calculator from WHO [23]. In addition to the UAE nationals, a sample of Arab non-nationals was selected with a ratio of nationals to Arab non-nationals of 2:1. Arab non-nationals included Syrian, Palestinian, Moroccan, Omani, Sudanese, Egyptian, Jordanian, Yemeni, Iraqi, Bahrain, Algerian, Lebanese, Tunisian, Saudi Arabian, Iranian, Libyan, and Somalian children.

 The protocol of the study along with the screening form, informed consent form for caregivers, recruitment form and the questionnaire were reviewed and approved by the Institutional Review Board (IRB) of the American University of Beirut (AUB), and by ethical authorities in the UAE including United Arab Emirates University (UAEU), Dubai Health Authority (DHA), UAE Ministry of Health and Prevention (MOHAP), and the University of Sharjah (UOS). All caregivers of study participants provided informed consent and were offered a 15-dollar book voucher as an incentive for their participation.

### Study population

Infants and toddlers were recruited from primary health care centers and outpatient clinics at hospitals between June 2019 – January 2020. A screening questionnaire was used to determine eligibility based on answers provided by the primary caregiver. To be eligible for the study, the infant or toddler must have been between 0 and 23.9 months of age with the absence of chronic illness, inborn errors of metabolism, and physical malformation that may affect the normal eating pattern and body composition. Non-Arab infants or infants of Arab non-nationals having resided in the UAE for less than three years were not eligible for the study.

### Data collection

Data were collected in Arabic. Prior to the initiation of data collection, all nutritionists were extensively trained to make sure that harmonized approaches were used in data collection, thus ensuring comparability of data between the various project sites. Interviewers were also trained to limit any judgmental verbal and non-verbal communication during the completion of the interview, to minimize social desirability bias. Additionally, a training manual was developed including detailed standard operating procedures (SOPs) to facilitate the data collection process. Trained nutritionists approached mothers in the waiting areas of the clinics, explained the study protocol and objectives, and invited them to participate. After receiving the written consent, the nutritionist collected the data in a one-to-one interview with the mothers, in a private room within the clinics. During this interview, the participating mothers completed a multi-component age-specific questionnaire that assessed socio-demographic and economic characteristics, early life feeding practices and food consumption, in addition to use of vitamins and mineral supplements. For the assessment of dietary intake, The Multiple Pass 24-hour recall was used. After collection, the data was entered into the SPSS statistical program for analysis.

A panel of experts, consisting of a public health nutritionist, a nutrition epidemiologist and pediatric nutritionist examined the developed questionnaire to ensure its content and face validity. The developed questionnaire was pilot tested on a convenient sample of 50 participants in order to ensure the clarity of the questionnaire as well as the cultural appropriateness of its content. The data generated from the pilot testing phase was not included in the analysis for this study. First developed in English, the questionnaire was also translated into the Arabic Language. In order to ensure parallel form reliability, the Arabic version of the questionnaire was back translated into English. The original and back translated English versions of the questionnaire were compared by a native English speaking expert to ensure accuracy and correctness.

Anthropometry was collected using a standardized protocol and included length, weight, and Mid-Upper Arm Circumference (MUAC) [24]. Collected data was evaluated using WHO Anthro Survey Analyser and MUAC criteria for malnutrition.

MUAC was measured using a calibrated plastic strip at the mid-point between the infant or toddler’s elbow and shoulder of the left arm with the arm being relaxed and hanging down the side of their body. MUAC was recorded to the nearest 0.1 cm. Length was measured to the last completed millimetre using the Seca 333 WLAN measuring rod for baby scales. To measure length, the measuring rod was placed on a raised, flat, and levelled surface. The infant was undressed (shoes along with any head accessories were removed) and the infant was placed lying down horizontally on the board. Using the left hand, the trained nutritionist held the infants’ legs leaving the right hand free to move the footboard. The infant’s head was positioned in a Frankfort Vertical Plane with the shoulders and hips aligned at right angles to the body. Minimal pressure was applied on the knees to straighten the infants’ legs and the soles of the feet flat on the footboard (the infant’s toes must be pointing up). The infant’s spine should not be arched when taking the measurement. The weight of infants was measured last as all clothing should be removed. Weight was measured using an electronic pan-type paediatric scale that is accurate to 0.1 kg (kg) (Seca 333I EMR ready Baby Scale). The infant or toddler was placed in the middle of the weighing surface and once the infant stopped moving, the weight was measured. To ensure that anthropometry was of high quality, measurements were repeated twice, and a maximal allowable difference was set. Any measurements beyond the set value were repeated.

Children’s Anthropometry was categorized based on the WHO growth standards classification [25].

**Table:** 

BMI status	BMI for age z-score (BAZ) for under five
Wasted	BAZ<-2
Normal	−2 ≤ BAZ≤+1
Possible risk of overweight	+1 < BAZ≤+2
Overweight	+2 < BAZ≤+3
Obese	BAZ>+3
Stunted if Height for age z-score (HAZ) <-2, not stunted if HAZ ≥-2	

MUAC assessment was based on WHO classification: Values <115 mm indicate severe acute undernutrition, values between 115 mm and 125 mm indicate moderate malnutrition [26]. Values were only established for infants and toddlers above 6 months of age. No cut-off points are available for infants less than 6 months.

Dietary intake was assessed using paper-based 24-hour recall Multiple Pass Food Recall 5-step approach. A booklet with household illustrations of cups, bowls, spoons, etc. was used during the 24-hour recall collection to help participants quantify the amount of food consumed (Additional file 1). The nutritionists obtained information about the name and time of each meal, weekday/weekend, location of food consumption, food consumed, portion size, preparation method, and the brand of the consumed food when applicable. A second 24-hour dietary recall was collected via phone interview for a randomly selected subsample (50%) of consenting participants to estimate within-person variance. Total water intake was recorded for the total day in a separate row in the 24 h-recall.

### Data flow, editing and processing

The dietetic professionals’ field supervisor, in the UAE, verified the 24 h-recalls to identify missing foods, unrealistic quantities reported, and called the mothers to obtain any missing information. Then, the 24 h-recalls were scanned and sent by email to the American University of Beirut where all foods and beverages were entered in English on the Nutritionist Pro software by a bilingual trained nutritionist [27]. Data in the questionnaires were entered into an electronic tablet application developed for the project and then verified by the UAE field supervisor for data quality and completion. Then the data was locked and uploaded to the server. Upon entering the information on the tablet, an automated email was sent to the AUB with the new completed survey. To link the 24 h-recall with the matching questionnaire from the same study participant, the picture of the first page of the 24-hr recall was scanned and uploaded to the corresponding questionnaire. A study ID was assigned to all participants.

Mixed dishes and traditional foods were added to the Nutritionist Pro software using single food items from obtained standardized recipes. The food composition table used was a combination of the USDA, food composition of the Middle Eastern region developed over the years by AUB, product packaging, or related websites when applicable, and studies published regarding UAE dishes [28, 29]. Within the Nutritionist Pro, the USDA database, edited by AUB for local main dishes, was selected for analysis. This allowed for the estimation of the intake of energy (kcal), carbohydrates, and total sugars in grams per day (g/day); and thereafter the calculation of free sugar (FS) intake (g/day) [30]. As data for lactose were available in the database, FS content was calculated by subtracting the lactose from the total sugars, based on the methodology of Yeung and Louie [31]. As per the definition of the WHO, sugars naturally present in unsweetened fruit juice, syrups, and honey were included in FS estimation [30, 32]. Additionally, added sugars in any form were included in the FS estimation [33]. Added sugars were calculated based on the 10-step methodology proposed by Kibblewhite et al., with a modification of the method from Louie et al., 2015 [34, 35]. Dietary reference intakes (DRIs) used were from the Institute of Medicine (IOM).

59% of infants and toddlers consumed supplements and while the type of supplement was collected from the caregiver (vitamin D, iron, multivitamin, calcium, zinc, vitamin C, and omega 3), around 56% of supplements’ consumers did not report the dose. To obtain an accurate record of nutrient intake among children, both dietary supplements (from the questionnaire) and foods and beverages (from the 24 h-recall) were considered. Thus, based on Paediatrician recommendations, dietary supplement brands commonly consumed, and brands available in the UAE, imputations on the dose of intake were carried out.

In this study, the estimation of breastmilk volume was carried out using the protocol of a previous FITS study for infants ≤ 12 months of age [36]. Infants under 6 months of age, who were exclusively breastfed, were considered to consume 780 milliliters (ml) of milk per day and those from 6 to 12 months as consuming 600 ml of milk per day (if no other milk was consumed). For all infants under 12 months, if any type of milk was consumed with the breastmilk, the volume of the other milk was deducted from the defined volume of breastmilk. As for toddlers above 12 months of age, the volume of milk was estimated according to the number of feeds per day. Toddlers between 12 and 18 months were estimated to consume 89 ml per feed, whereas toddlers above 18 months consumed 59 ml per feed. Data for breastfeeding were analysed according to Infant and Young Child Feeding Indicators – WHO classification [37] as below:

- Children ever breastfed: Children born in the last 24 months who were ever breastfed/ Children born in the last 24 months.
- Exclusive breastfeeding under 6 months: Infants 0–5 months of age who received only breast milk during the previous day/ Infants 0–5 months of age.
- Continued breastfeeding at 1 year: Children 12–15 months of age who received breast milk during the previous day/ Children 12 – 15 months of age.
- Continued breastfeeding at 2 years: Children 20–23 months of age who received breast milk during the previous day/ Children 20 – 23 months of age.
- Introduction of solid or semi-solid food: Infants 6–8 months of age who received solid, semi-solid, or soft foods during the previous day/ Infants 6 – 8 months of age.

### Data Analysis

The estimated usual intake was calculated using the PC-side software produced by Iowa State University in 2001 [38]. This software allows for estimation of the distribution of usual nutrient intakes, foods consumed almost daily, and other dietary components. The usual intake calculated was based on nutrient intake from food sources (including the second day 24-hour recalls) and was then adjusted to take into account nutrient intakes from dietary supplements using the combined method [39]. Distribution of intakes and the probability of exceeding AI (%>AI) and UL (%>UL) and meeting the EAR (%<EAR) were produced from the usual intake. The EAR was used to assess nutrients’ dietary adequacy, whereas AI was used for nutrients with no established EAR value. As for the macronutrients, the acceptable macronutrient distribution range (AMDR) was used to measure the macronutrient intake of toddlers over 12 months of age. Adherence to food group recommendations was done for toddlers above 12 months of age based on the AHA/ AAP 2005 dietary recommendations for children and adolescents [40]. Limited quantitative food group recommendations have been established for infants ages 0 – 11.9 months. Data analysis was carried out using Statistical Package for Social Sciences 25.0 (SPSS for Windows, 2013, Chicago: SPSS Inc.) and the level of significance was set at *p* < 0.05. Frequencies and proportions as well as means ± SE were used for categorical and continuous variables respectively. The Chi-squared test was used to compare categorical variables whereas independent t-tests were used to compare continuous variables.

## Results

### Household characteristics of study population

The study sample was comprised of 276 infants and toddlers between the age of 0 and 23.9 months including 180 UAE nationals and 96 Arab non-nationals. Sociodemographic and household characteristics of the study population are shown in Table 1. The mean age of the sample was 8.8 months. The sample included approximately 55% of males and 45% females. The unemployment rate was higher in mothers (71.7%) compared to fathers (2.9%) with no significant difference between nationals and Arab non-nationals. In terms of education, a significantly higher percentage of Arab non-nationals’ mothers and fathers (65%) had a university or graduate school degree as compared to approximately 45% of nationals (*p* = 0.001). A significant difference was also seen in house ownership between nationals and Arab non-nationals where 65% of nationals owned the house they currently lived in as compared to 12% of Arab non-nationals (*p *= <0.001). 15% of nationals had a crowding index below1, whereas only 3% of Arab non-nationals fell in this category. As for rates of exclusive breastfeeding, 46% of infants aged 0-3.9 m were exclusively breastfed at 4 months and 37% at 6 months of age, with nationals having a lower rate of exclusive breastfeeding at 6 months (~35%) as compared to Arab non-nationals (~43%) although the difference did not reach statistical significance.

**Table 1:** Characteristics of UAE Infants and Toddlers ages 0 to 23.9 months FITS 2020 (*n* = 276)

Characteristics	Total (*n* = 276)	Nationals (*n* = 180)	Arab non-Nationals (*n* = 96)	*P*-value
Mean ± SE				
Age (in months)	8.8 ± 0.4	8.3 ± 0.5	9.6 ± 0.7	0.11
n (%)				
Gender				0.06
Male	151 (54.7)	91 (50.6)	60 (62.5)	
Female	125 (45.3)	89 (49.4)	36 (37.5)	
Age				
0 – 3.9 months	82 (29.7)	60 (33.3)	22 (22.9)	0.16
4 – 5.9 months	31 (11.2)	23 (12.8)	8 (8.3)	
6 – 8.9 months	46 (16.7)	25 (13.9)	21 (21.9)	
9 – 11.9 months	27 (9.8)	16 (8.9)	11 (11.5)	
12 – 23.9 months	90 (32.6)	56 (31.1)	34 (35.4)	
Exclusive Breastfeeding at 4 months (among 0-3.9 m)	38 (46.3)	27 (45.0)	11 (50.0)	0.69
Exclusive Breastfeeding at 6 months (among 0-5.9 m)	42 (37.2)	29 (34.9)	13 (43.3)	0.42
Education of Mother				0.001*
Up to Secondary^a^	132 (47.8)	99 (55.0)	33 (34.4)	
University/Graduate Degree	144 (52.2)	81 (45.0)	63 (65.6)	
Education of Father				0.001*
Up to Secondary^a^	133 (48.4)	100 (55.6)	33 (34.7)	
University/Graduate Degree	142 (51.6)	80 (44.4)	62 (65.3)	
Employment Status of Mother				0.60
No	198 (71.7)	131 (72.8)	67 (69.8)	
Yes	78 (28.3)	49 (27.2)	29 (30.2)	
Employment Status of Father				1.00
No	8 (2.9)	5 (2.8)	3 (3.2)	
Yes	267 (97.1)	175 (97.2)	92 (96.8)	
Crowding Index				0.01*
<1	30 (11)	27 (15.3)	3 (3.1)	
1-2	169 (62.1)	106 (60.2)	63 (65.6)	
≥2	73 (26.8)	43 (24.4)	30 (31.3)	
House Ownership (*n* = 242)^b^				<0.001*
Not owned	131 (54.1)	55 (35.3)	76 (88.4)	
Owned	111 (45.9)	101 (64.7)	10 (11.6)	
Dietary Supplement Use^c^				0.67
No	114 (41.3)	76 (42.2)	38 (39.6)	
Yes	162 (58.7)	104 (57.8)	58 (60.4)	

*Indicates significance at *p* < 0.05

^a^Up to secondary school includes: No schooling, primary school, intermediate school, high school, or technical diploma

^b^House ownership has a different sample size of 242 as 34 participants did not answer the question

^c^Dietary supplement use included taking any multivitamin, vitamins, or minerals (chewable, tablets or drops)

Percent population by Nationality: Nationals 65%; Arab non-nationals 35%

### Nutritional status

**Fig. 1:**
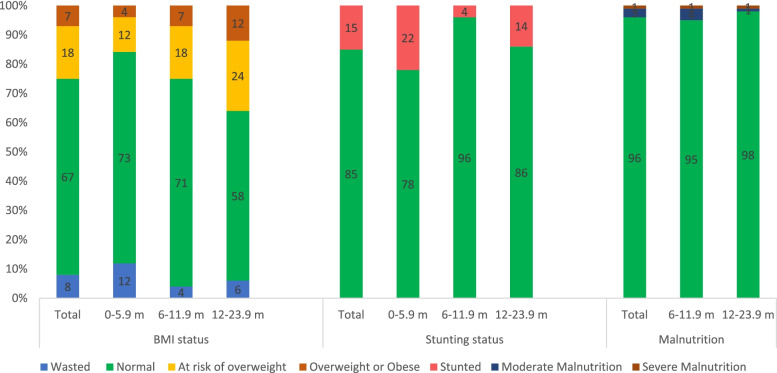
Malnutrition amongst UAE Infants and Toddlers ages 0 to 23.9 months (%) FITS 2020 (*n* = 276). MUAC reference values have not been established for infants 0 – 5.9 months, only infants and toddlers above 6 months were considered. No significant difference was observed between Nationals and Arab non-nationals

As shown in Fig. 1, approximately 15% of all infants and toddlers were stunted ranging from 22% to 0 – 5.9 months to 14% in 12 – 23.9 months old. A higher prevalence of stunting was observed among nationals (18%) as compared to Arab non-nationals (9%) with no statistical significance (data not shown). According to MUAC, a total of 2.5% of infants and toddlers, above 6 months of age, were moderately malnourished and 1.2% were severely malnourished (Fig. 1). As for the BMI status, 8% of infants and toddlers were wasted with the highest prevalence among infants 0 – 5.9 months (12%). Approximately 18% of infants and toddlers were at risk of being overweight with an increased rate from around 13% 0 – 5.9 months to 18% among 6 – 11.9 months to almost 25% in the age group 12 – 23.9 months old. 7% of infants and toddlers were overweight and obese with a higher rate observed within the age range 12 to 23.9 months (12%). Among those who are at risk of overweight, overweight, and obese, 20% were stunted versus 13% stunted among children with normal BMI status (data not shown). No statistical difference was noted between nationals and Arab non-nationals nutritional status.

### Feeding practices

**Fig. 2:**
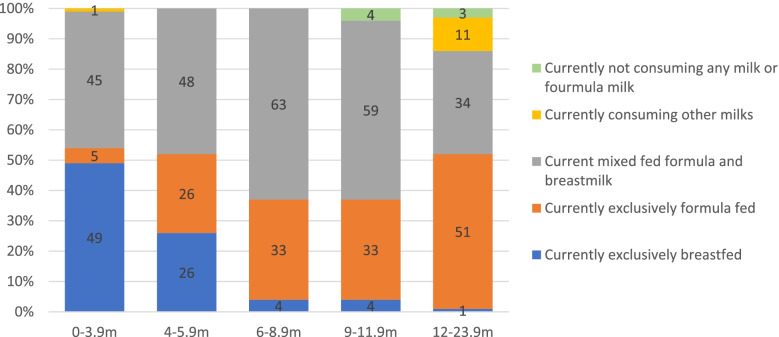
Different Feeding Practices of Infants and Toddlers ages 0 – 23.9 months (%) FITS 2020 (*n* = 276)

Feeding practices of infants and toddlers, in five age groups (0 – 3.9 m, 4 – 5.9 m, 6 – 8.9 m, 9 – 11.9 m, and 12 – 23.9 m) are presented in Fig. 2. Exclusive breastfeeding was noted in 45% of infants aged 0 to 3.9 months. This percentage declined to 26% at 4-5.9 m, 4% at 6-11.9 m and only 1% of the sample being exclusively breastfed above 12 m of age. Mixed feeding of formula and breastmilk was observed in 45% of infants aged 0-3.9 m which increased to 48% at 4-5.9 m, reaching a peak of 63% at 6-8.9 m, and declining to 59% in 9-11.9 reaching 34% in infants above one year of age. Exclusively formula feeding was observed in 5% of 0-3.9 m infants with an increasing trend to 26% in 4-5.9%, rising to 33% at 6-11.9 m and reaching a peak of 51% at 12-23.9 m. Of the total sample, approximately 95% of infants were ever breastfed (data not shown).

### Complementary feeding

**Fig. 3:**
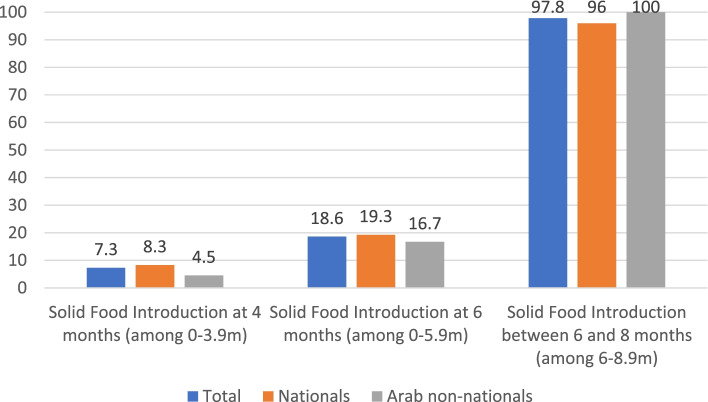
Introduction of complementary foods to UAE Infants and Toddlers ages 0-23.9 (%) FITS 2020 (*n* = 276)

Approximately 7% and 19% of infants were introduced to complementary foods before 4 and 6 months of age respectively. Most children (96-100%) were introduced to complementary foods at 6 months of age. No significant difference was noted between nationals and Arab non-nationals with regards to the introduction of complementary food (Fig. 3).

**Fig. 4:**
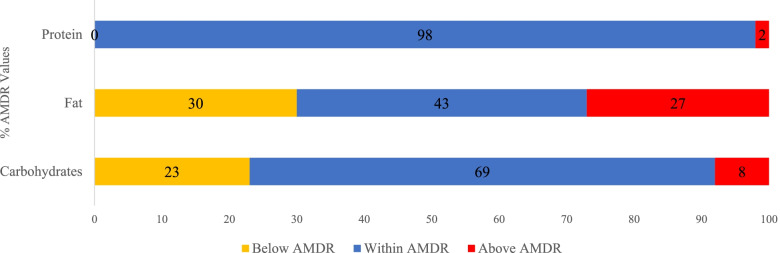
Toddlers aged 12 to 23.9 months meeting Macronutrient AMDR FITS 2020 (*n* = 90). The AMDRs among 12-23.9 months children are as follows: Protein: 5-20%, Fat: 30-40%, Carbohydrate: 45-65%

In assessing the dietary adequacy of infants and toddlers, macronutrient and micronutrient intakes were assessed. Regarding macronutrient intake, most toddlers were consuming within the AMDR except for fat where 27% exceeded AMDR for fat (Fig. 4). A total of 23% did not consume recommended AMDR of carbohydrates, while 8% were consuming above AMDR of carbohydrates. As for the percentage of children aged 0 to 2 years exceeding the ESPGHAN cut-off for free sugars, set at a maximum of 5% of energy intake, data showed that 28.6% had excessive intakes overall, 10% in 0-5.9, 21.9% in 6-11.9 and 56.7% in 12-23.9 month [41]. Percent of children consuming above the adequate intake of linoleic acid and alpha-linolenic acid was noted in approximately 32% and 35% of 0 – 5.9 m old, and 34% and 28% of 12 – 23.9 m old respectively. Only 28% of toddlers aged 12 – 23.9 months consumed above 19 g/d of fiber.

The total usual intake of micronutrients was estimated using both food intake from dietary recall and micronutrient intake from supplements (Table 2). Among 0 – 5.9-month-old children, a low percentage of infants met the zinc AI (23%). The main micronutrient inadequacies among 6-11.9 months old children were iron and zinc where approximately 47% and 32% of infants were consuming below EAR respectively. In this age group a low percentage of children consumed above AI for vitamin D (60%), and vitamin A (59%). As for toddlers ages 12 – 23.9 m, intakes below the EAR for niacin (21%), folate (21%), vitamin D (49%), phosphorus (17%), calcium (36%), and vitamin B6 (10%) were noted.

**Table 2:** Usual Energy and Nutrient Intakes from food and dietary supplements among Infants and Toddlers *0-23.9 months* in FITS UAE 2020

	0 – 5.9 months (*n*=113)			6 – 11.9 months (*n*=73)				12 – 23.9 months (*n*=90)			
Nutrient Intake	Mean ± SE	% DRI Compliance		Mean + SE	% DRI Compliance			Mean + SE	% DRI Compliance		
		>AI	>UL		>AI	<EAR	>UL		>AI	<EAR	>UL
Energy, kcal/d	667.8 ± 17.5	--	--	840.5 ± 31.5	--	--	--	1001.7 ± 37.1	--	--	--
Fat, g/d	38.4 ± 0.7	94.7	--	36.6 ± 1.6	68.5	--	--	39.2 ± 1.7	--	--	--
Saturated Fat, g/d	15.5 ± 0.4	--	--	12.5 ± 0.8	--	--	--	13.7 ± 0.7	--	--	--
Cholesterol, mg/d	92.7 ± 3.3	--	--	85.1 ± 9.3	--	--	--	124.1 ± 9.1	--	--	--
Monounsaturated Fat, g/d	12.6 ± 0.4	--	--	10.3 ± 0.8	--	--	--	11.6 ± 0.6	--	--	--
Polyunsaturated Fat, g/d	4.7 ± 0.2	--	--	4.7 ± 0.3	--	--	--	6.2 ± 0.4	--	--	--
Linoleic acid, g/d	4.6 ± 0.2	31.9	--	5.3 ± 0.3	50.7	--	--	6.2 ± 0.4	34.4	--	--
α Linolenic acid, g/d	0.5 ± 0.0	35.4	--	0.5 ± 0.0	46.6	--	--	0.6 ± 0.0	27.8	--	--
Carbohydrates, g/d	66.1 ± 2.4	51.3	--	103.5 ± 4.1	58.9	--	--	129.3 ± 5.3	--	30.0	--
Total Sugar, g/d	54.5 ± 1.8	--	--	49.9 ± 3.4	--	--	--	54.5 ± 3.0	--	--	--
Free Sugar, g/d	4.7 ± 1.3	--	--	9.8 ± 2.1	--	--	--	18.1 ± 1.5	--	--	--
Free Sugars, % energy ^a^	2.3 ± 0.5	--	10	4.4 ± 0.7	--	--	21.9	7.4 ± 0.5	--	--	56.7
Added Sugars, g/d	0.6 ± 0.2	--	--	5.1 ± 0.8	--	--	--	13.6 ± 1.4	--	--	--
Protein, g/d	13.2 ± 0.6	89.4	--	21.8 ± 1.0	--	5.5	--	32.9 ± 1.5	--	1.1	--
Dietary Fiber, g/d*	0.1 ± 0.0	--	--	1.7 ± 0.3	--	--	--	17.7 ± 2.6	28.1	--	--
Vitamin C, mg/d	60.1 ± 2.8	96.5	--	71.1 ± 3.8	71.2	--	--	66.1 ± 3.5	--	2.2	0.0
Thiamine, mg/d	0.4 ± 0.0	65.5	--	0.7 ± 0.0	94.5	--	--	0.8 ± 0.0	--	6.7	--
Riboflavin, mg/d	0.7 ± 0.0	98.2	--	1.0 ± 0.1	93.2	--	--	1.2 ± 0.1	--	3.3	--
Niacin, mg/d*	3.5 ± 0.3	70.6	--	6.0 ± 0.5	63.6	--	--	10.4 ± 0.8	--	21.4	--
Vitamin B6, mg/d	0.2 ± 0.0	99.1	--	0.6 ± 0.0	91.8	--	--	0.8 ± 0.0	--	10.0	0
Folate, μ/d	94.5 ± 7.0	49.6	--	176.0 ± 10.6	87.7	--	--	244.1 ± 13.0	--	21.1	--
Vitamin B12, μ/d	0.9 ± 0.1	96.5	--	1.2 ± 0.1	80.8	--	--	2.0 ± 0.2	--	10.0	--
Vitamin D, μ/d	10.8 ± 0.7	69.0	5.3	12.2 ± 0.9	60.3	--	0.0	10.2 ± 0.8	--	48.9	0.0
Vitamin A RAE**	584.6 ± 14.6	98.2	15.9	596.8 ± 32.3	58.9	--	13.7	505.5 ± 27.7	--	8.9	17.8
Vitamin K, μ/d*	10.8 ± 2.1	85.0	--	25.2 ± 3.2	98.6	--	--	32.3 ± 3.3	36.7	--	--
Calcium, mg/d	427.9 ± 22.6	99.1	5.3	571.1 ± 32.9	95.9	--	1.4	649.4 ± 33.2	--	35.6	0.0
Phosphorus, mg/d*	214.1 ± 11.0	89.1	--	384.1 ± 23.5	67.0	--	--	703.7 ± 39.4	--	16.6	0.0
Iron, mg/d	4.0 ± 0.5	99.1	0.0	10.3 ± 1.0	--	46.6	1.4	8.9 ± 0.7	--	11.1	0.0
Zinc, mg/d	2.2 ± 0.2	23.0	12.4	3.7 ± 0.3	--	31.5	21.9	4.4 ± 0.3	--	16.7	11.1
Sodium, mg/d	240.9 ± 16.6	99.1	--	445.7 ± 31.2	76.7	--	--	920.5 ± 56.9	45.6	--	25.6
Potassium, mg/d	620.1 ± 26.7	98.2	--	1047.3 ± 47.5	82.2	--	--	1364.3 ± 61.1	10.0	--	--

*Note: Estimated usual intakes are calculated from the PC-side software. Values are presented as percentages of DRI compliance based on the total usual intakes

**The vitamin A upper level is based on preformed Vitamin A

*DRI* Dietary Reference Intake, *AI* Adequate Intakes, *UL* Tolerable Upper Intake level, *EAR* Estimated Average Requirement

SOURCES: WHO, Dietary Reference Intakes for Calcium, Phosphorous, Magnesium, Vitamin D, and Fluoride (1997); Dietary Reference Intakes for Thiamine, Riboflavin, Niacin, Vitamin B6, Folate, Vitamin B12, Pantothenic Acid, Biotin, and Choline (1998); Dietary Reference Intakes for Vitamin C, Vitamin E, Selenium, and Carotenoids (2000); and Dietary Reference Intakes for Vitamin A, Vitamin K, Arsenic, Boron, Chromium, Copper, Iodine, Iron, Manganese, Molybdenum, Nickel, Silicon, Vanadium, and Zinc (2001); Dietary Reference Intakes for Water, Potassium, Sodium, Chloride, and Sulphate (2005); and Dietary Reference Intakes for Calcium and Vitamin D (2011)

^a^Free sugars is compared to the recommended value of below 5%. Participants consuming above 5% were considered consuming above the UL

Across all age groups (0-5.9, 6-11.9, 12-23.9 m), the UL was exceeded for zinc (12%, 22%, and 11% respectively) and vitamin A (16%, 14%, and 18% respectively). In the age group 0 – 5.9 m, approximately 5% had usual intakes above UL for vitamin D. Among infants aged 0 – 11.9 months, roughly 1 – 5% exceeded the UL for calcium. 26% of toddlers over 12 months of age exceeded the chronic disease risk reduction (CDRR) for sodium. No significant differences were noted between nationals and Arab non-nationals.

### Food groups

**Fig. 5:**
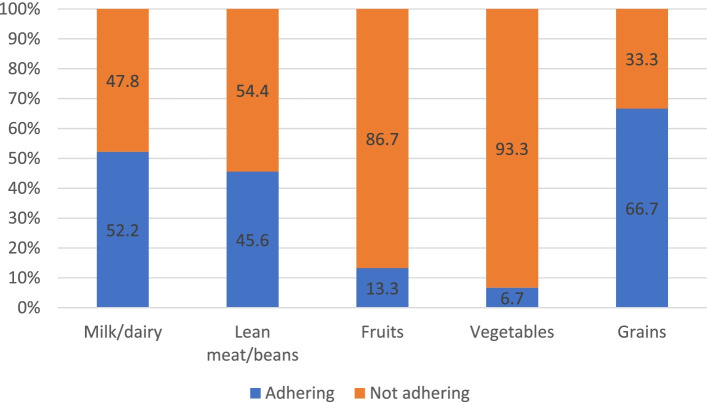
Adherence to dietary recommendations pertinent to food group intake, in children above one year of age

Adherence to dietary food groups recommendations for toddlers ages 12 to 23.9 is summarized in Fig. 5. In age group 12 – 23.9 months, a high percentage of toddlers (87% and 93%) were not adhering to fruits and vegetable recommendations of 240 and 180 g respectively. Approximately 33% of toddlers did not follow the recommendations for grains (57 g) and 54% for lean meat and beans (43 g). As for milk and dairy, 48% of toddlers did not adhere to dietary recommendations of 2 cups of milk/dairy per day.

## Discussion

This study is the first to examine the nutritional status and adequacy of feeding practices of a representative sample of infants and toddlers residing in the three major Emirates in the UAE, of Arabic (non-UAE) and UAE nationalities. The study underscores the double burden of malnutrition in the country and the poor feeding practices of infants and toddlers which could further compromise young children’s health status and exacerbate NCDs in the country.

The findings of this study demonstrated a high prevalence of stunting (15%) in comparison to other countries in the region where stunting among children below 5 years of age was shown to be 7.8% in Jordan, and 6.4% in Kuwait [42]. The study also revealed a high prevalence of overweight and obesity (7%), particularly among toddlers ages 12 to 23.9 months (12%) in comparison to the other countries where the prevalence of overweight and obesity was 4.7% in Jordan and 5.5% in Kuwait [42]. The childhood nutrition status is strongly correlated with the development of NCD such as diabetes and CVD in later adulthood along with poor potential in later life [8]. Moreover, childhood obesity profoundly affects a child’s physical, social, and emotional well-being and academic performance [43].

Several factors play a role in the prevalence of obesity such as environmental, lifestyle, and cultural factors. In economically emerging countries with high Gross Domestic Product (GDP) like the UAE, the co-occurrence of under and overnutrition is thought to result from the slow disappearance of undernutrition and the rapid shift in food consumption patterns towards high caloric fatty foods of poor nutritional value [44]. This nutrition transition phenomenon from traditional foods rich in fruits, vegetables, and dairy products (precisely yogurt) to a more westernized diet has been attributed to urbanization, modernization, and the rapid demographic and epidemiologic shifts, which are accompanied by poorer infant and young child feeding practices, fast-paced, technology driven and stressful lifestyles and lower physical activity levels.

Malnutrition in all its forms is a significant public health challenge incurring enormous costs on healthcare systems and societies around the world. One of the main variables directly associated with under and overnutrition, which is highlighted in this study, is the inadequate breastfeeding and infant feeding practices of the UAE population. This study showed that most women (95%) initiated breastfeeding at birth which is similar to a previous study conducted among mothers in Abu Dhabi, UAE (95.6%) [18] and other countries in the region that found a high prevalence of ever breastfed infants such as 98% in Oman, 94% in Qatar, 93% in Jordan, and 96% in Egypt [45]. Despite the high prevalence of breastfeeding initiation, the duration of exclusive breastfeeding falls short of recommendations. In the UAE, approximately only 37% of infants were exclusively breastfed at 6 months, which is considered to be ‘fair’ according to the WHO IYCF rating [45]. The UAE exclusive breastfeeding rate for infants 0 – 6 months falls noticeably below the WHO target of 60% by 2025 indicating need for special attention [42]. Although exclusive breastfeeding results were not optimal, UAE had a higher rate compared to neighbouring countries such as Turkey (30%), Qatar (29%), Oman (23%), and Jordan (25%) [8]. Moreover, according to WHO indicators, this study shows ‘poor’ levels of sustained breastfeeding as few infants were breastfed at 1 year and 0% were breastfed at 2 years of age [45]. Breastfeeding has a well-established crucial role in the first 1,000 days of life as it is the ultimate source of nutrition for infants, promotes sensory and cognitive growth and development, and provides infants with protection against infectious and chronic diseases that extends into adulthood [46]. Moreover, studies have shown that infants who were breastfed for longer durations were less likely to become overweight and obese in their childhood years, performed better in school, and had higher IQ [42]. Breastfeeding is also beneficial to maternal health, birth spacing, as well as the widely recognized economic advantages [47]. Possible attributable factors impacting the duration and willingness to breastfeed is the economic and demographic changes that transitioned the UAE into a multinational, urbanized and modern society, concurrent with a steep rise in women pursuing higher education and joining the workforce [47–50]. In the current study, the factors related to early termination of breastfeeding were related to claims of poor milk supply and challenges faced when going back to work. It is noteworthy that, as per the maternity leave policy, mothers in the UAE are entitled to 90 days of absence in addition to reduced working hours by 2 h per day during the first 4 months from the date of resuming work [51]. In the UAE, efforts have been made to support breastfeeding such as the Sharjah Baby-Friendly Campaign launched in 2012 which developed breastfeeding education resources and conducted breastfeeding promotion seminars in health facilities and community [52].

Among infants who were not exclusively breastfed for 6 months, around 48% were mixed fed (infant formula and breastmilk) and 26% were exclusively formula-fed. This is concerning as breastfeeding has long been documents as the normative method for feeding and promoting the optimal development of infants.

The timely introduction of complementary food in childhood and their dietary adequacy are cornerstones for lifelong health that also influence the risk for malnutrition [8]. According to the WHO, timely introduction of complementary food is at 6 months of age where breastmilk is no longer sufficient to meet the energy and nutrient needs of the growing infant [53]. It was evident, in the current study, that the timely introduction of solid food was ‘very good’ according to the WHO IYCF rating [45]. Likewise, Abdulrazzaqin el al. reported that 90% of Emirati infants in Dubai and Al-Ain, UAE, had been introduced to complementary foods between 5 and 6 months [17]. This is critical, as early weaning onto solid foods, before 6 months of age, has been found to cause allergies, suboptimal growth and increase the risk of developing obesity and CVD later in adulthood, primarily due to the replacement of breastmilk with foods of poor nutritional value [54]. Nevertheless, other studies have shown that a very late introduction of solid food (beyond 6 – 8 months of age) may result in a different set of health problems related to micronutrient deficiencies such as iron deficiency anaemia, blindness, and mortality due to vitamin A deficiency, irreversible brain damage due to iodine deficiency and rickets due to vitamin D deficiency [55, 56].

With regards to macro and micronutrient adequacies, results of this study indicate an overconsumption of fat, low intake of essential fatty acids across all age groups, and both deficient and excess intake of vitamins and minerals across all age groups. Although a substantial portion of toddlers were exceeding the AMDR for fat (27%), the consumption of the essential fatty acids, linolenic and linoleic acid, was low across all age groups. These essential fatty acids have a critical role in the development of the child’s cognition and nervous system [57]. In the current study, toddlers were consuming within the AMDR range for proteins indicating adequate intake. This is of importance as low consumption of protein, energy, and micronutrients cause protein-energy malnutrition (PEM). PEM is a common fatal childhood disorder characterized by wasting, stunting, and edematous malnutrition [39].

As for the intake of free sugar, the current study revealed intakes above the recommendation among 28% of toddlers 12 -23.9 m of age. This is higher than the United States (US), where a high rate of childhood obesity exists, whereby 13.9% of children consumed above the recommendation [58]. This excess intake during infancy is alarming as consumption of free sugar displaces vital nutrients and leads to obesity in later childhood [32]. Moreover, consuming dietary free sugars at high frequency is one of the main risk factors for early childhood caries (ECC), which is an international public health challenge especially amongst young children and it is highly prevalent in the UAE [59].

As for micronutrient intake, this study revealed an inadequate intake of vitamin D as around 50% toddlers had intakes below EAR. Results are in line with other regional countries such as Lebanon and Saudi Arabia which have a range of 45-62% prevalence of vitamin D deficiency among children [60]. Inadequate vitamin D intake is also a global concern as inadequate intake among infants and toddlers was also noted in the US, Canada, and the United Kingdom [61–63]. Interestingly, although 53% of children were consuming vitamin D supplements, a substantial percent was still below the EAR. Vitamin D deficiency is a risk factor for infants and toddlers, and is associated with the development of rickets, growth failure, predisposition to respiratory infections in infancy, and lethargy [64–67]. The peak incidence of the development of rickets is between the age 3 and 18 months which can be prevented by adequate dietary intake [68].

Dietary adequacy of infants and toddlers in the UAE further revealed a low intake of iron across all age groups. Infants and toddlers (specifically above 6 months of age) are at high risk of developing iron deficiency anaemia as their rapid growth will result in an exhaustion and depletion of the infants’ iron stores [69]. In fact, studies have shown that iron-deficiency anaemia is the most prevalent nutritional problem among children in the MENA region [60]. Iron deficiency during infancy increases the risk of mortality, impaired cognitive and psychomotor development, defect in behavioural development leading to poor school performance and decrease productivity in later life [70, 71]. As iron deficiency is common among children globally, it has been suggested by the American Academy of Paediatrics that breastfed infants be supplemented with liquid iron supplementation until iron fortified infant cereals may be introduced [72, 73].

Vitamin A intake was below EAR in approximately 9% of toddlers ages 12 – 23.9 months. Vitamin A deficiency is a public health problem for children in many areas, and is prevalent in 10% of children ages 6 – 59 months in the Middle East [74]. Vitamin A deficiency is correlated with irreversible blindness and high mortality rate. The WHO suggests that supplements be delivered to children ages 6 to 59 months twice yearly in order improve child survival and health [75].

It is pivotal to mention that the current study assessed micronutrient intake from both food and supplements. Thus, intakes of some vitamins and minerals were reported to reach above UL for infants and toddlers, such as the case of preformed vitamin A whereby 14 to 18% of infants and toddlers exceeded its UL. This may possibly be due to the combined consumption of vitamin A from supplements and fortified foods such as milk and oil that have been fortified in the UAE in an attempt to lower the prevalence of vitamin A deficiency [76]. High intakes of vitamin A among infants and toddlers may lead to nausea, vomiting, fever, headaches, and bulging fontanelle.

Moreover, of concern in the current study is intakes of zinc above the UL which is possibly due to the combined contribution of supplements and food sources. High intakes of zinc may result in anaemia and impaired immune function among young children [77]. However, it is worth mentioning that the zinc UL is based on limited data and has been previously criticized for the value given.

An inadequate intake of potassium, with 90% of children ages 12 – 23.9 months consuming below the AI was evident in this study which could be attributed to the low vegetable’s consumption observed in this age group. A concurrent high intake of sodium above UL (among 30% of children) was also observed in this age group. A meta-analysis of 18 experimental and observational studies showed that for every additional gram of sodium intake per day, systolic blood pressure increase by 0.8 mmHg and diastolic blood pressure by 0.7 mmHG [78]. Additionally, there was a strong association between sodium intake and blood pressure among overweight children and children with a low potassium intake [78]. Thus, a low potassium/high sodium ratio is concerning as it correlates with higher blood pressure during childhood which can be predictive for hypertension in adulthood [79].

Considering the AHA/AAP food group recommendations for toddlers 12 – 23.9 m old, an alarming observation in the current study is the very low consumption of fruits and vegetables among toddlers as they fall far below the recommendations. Low intake of fruits and vegetables is concerning as intake of fruits and vegetables are correlated with chronic diseases and a higher intake during childhood translate into healthier dietary habits in adulthood [80]. In addition, a substantial portion of toddlers (48%) had a low consumption of dairy products. This is also a major concern as milk and dairy products are major sources of calcium in children’s diet which is pivotal for proper growth and development. In the first 2 years of life, it is critical that calories being consumed for proper growth and development are quality calories and the food patterns being built are healthy to prevent childhood obesity and NCD in later life [81].

In general, this study characterizes young children’s diets as deficient in iron-rich foods with the need of improvement in fat and carbohydrate quantity and quality and underscore the double burden of malnutrition among infants and toddlers in the UAE. The current practices of breastfeeding and infant feeding in the UAE are similar to findings from other countries in the region and reflect the rapid nutrition transition witnessed in the region [50, 82]. The findings highlight the issues that need to be tackled in child feeding practices and alert policy makers for the need of evidence-based interventions to improve dietary practices in the UAE in line with international standards.

An important limitation of this study is the use of 24-HR recalls which poses a risk of reporting bias and errors remembering breastfeeding practices, particularly among mothers with older children [83]. Moreover, only the three major emirates were included in the present study which may limit the generalizability of the study results across the UAE. Additionally, the cross-sectional study design does not allow the investigation of causality which may limit evaluation of cause-effect relationships. Despite these evident limitations, this study provides vital and new information about infant and young children feeding practices based on a representative sample from three major emirates in the UAE that host around 85% of the country’s population. The study allows the comparison of dietary intake among UAE children with international recommendations. Currently, the UAE are adopting the recommendations from the WHO, however, more efforts should be made to ensure proper implementations of the guidelines.

## Conclusions

In conclusion, a triple burden of malnutrition exists in the UAE, with high levels of stunting, wasting overweight, and micronutrient deficiencies/excesses among infants and toddlers. The adequate consumption of food groups lags behind international recommendations. These findings highlight the need for further research to identify the cultural barriers to improve infant feeding practices, most importantly the exclusive breastfeeding for the first 6 months of life. The data, provided in this study, offer essential information that may guide the development of national strategies to promote, protect and enforce proper infant feeding practices. According to the current study results, special attention should be given to promote exclusive breastfeeding for 6 months and regulation of intake of supplements specifically for vitamins A, D, and iron, increasing the intake of fruits and vegetables, dairy products, whole grains and decreasing the intake of free sugars.

## Supplementary Information

**Additional file 1.**

## Data Availability

The datasets used and/or analysed during the current study are available from the corresponding author on reasonable request.

## Abbreviations

AI: Adequate Intake
AMDR: Acceptable macronutrient distribution range
EAR: Estimated average requirement
FITS: Feeding Infants and Toddler Study
IYCF: Infant and Young Child feeding practices
MUAC: Middle-upper arm circumference
UAE: United Arab Emirates
UL: Tolerable upper level
WHO: World Health Organization
