# Supplementary material for: Nutritional status and adequacy of feeding Practices in Infants and Toddlers 0-23.9 months living in the United Arab Emirates (UAE): findings from the feeding Infants and Toddlers Study (FITS) 2020
Source: BMC Public Health. 2022 Feb 15;22:319. doi: 10.1186/s12889-022-12616-z (PMC8848814; doi:10.1186/s12889-022-12616-z)
Supplement: Supplementary file 1 — Additional file 1. [file 12889_2022_12616_MOESM1_ESM.doc]

## Additional file 1:

## Two-dimensional chart (portion sizes) used in the 24-hr recall for the study


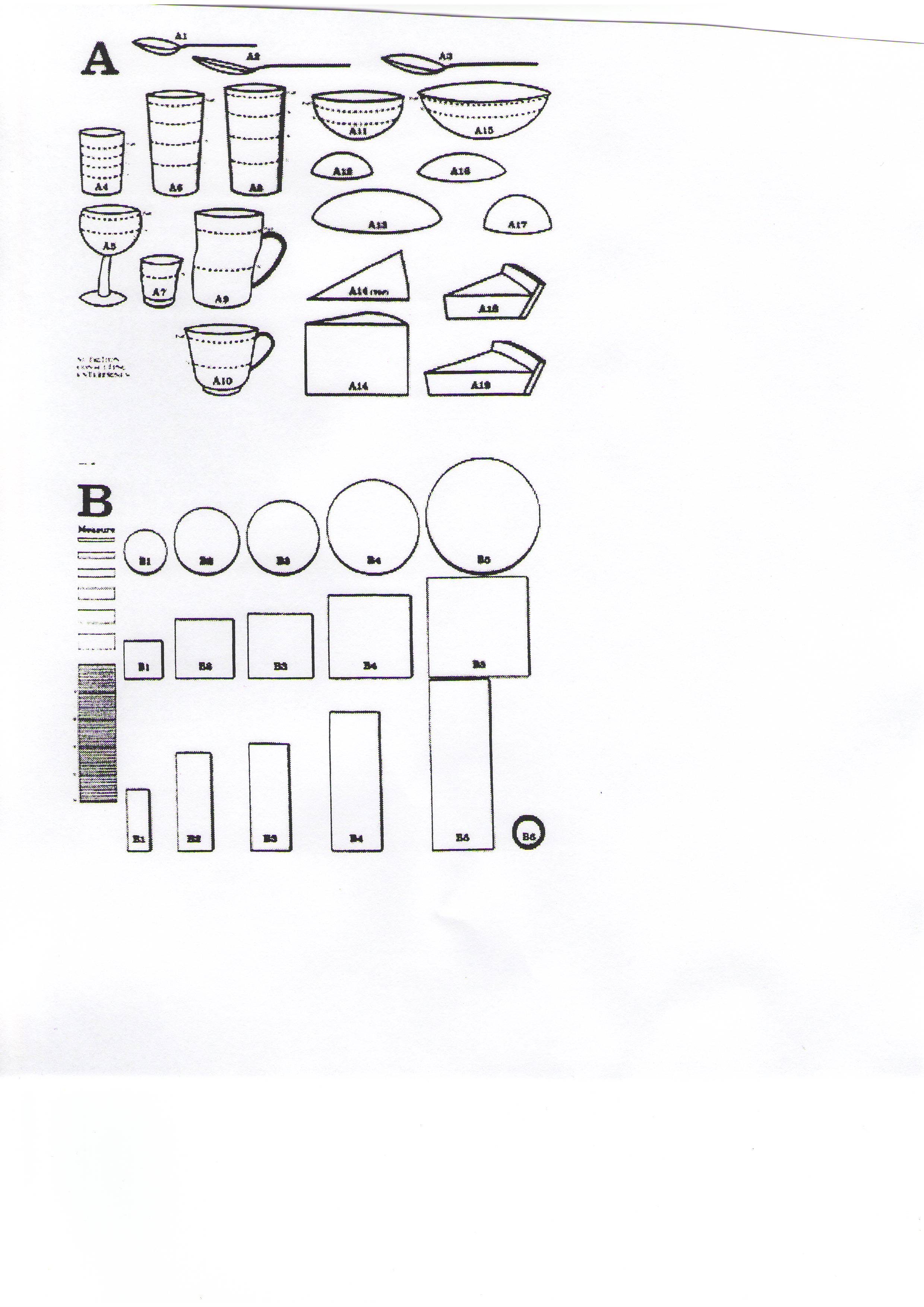


**Reference: Millen B and Morgan JL. The 2D Food Portion Visual. Farmingham, MA: Nutrition Consulting Entreprises,1996**
